# Intramuscular Myxoma of the Penis: A Case Report

**DOI:** 10.7759/cureus.101646

**Published:** 2026-01-15

**Authors:** Paul E Ngwu, Ifeanyichukwu E Ihedoro, Chibueze P Ohiarah, Emeka S Ogbata, Enyinnanya V Onyemachi

**Affiliations:** 1 Urology, Federal Medical Centre, Umuahia, Umuahia, NGA; 2 Urology, Eleos Specialist Hospital, Umuahia, NGA; 3 Urology, North Cumbria Integrated Care NHS Foundation Trust, Carlisle, GBR; 4 Surgery, Federal Medical Centre, Umuahia, Umuahia, NGA; 5 Anatomic Pathology, Federal Medical Centre, Umuahia, Umuahia, NGA

**Keywords:** intramuscular, myxoma, penile, rare tumours, tumour

## Abstract

Intramuscular myxoma (IMM) is a rare benign mesenchymal tumour, characterised by hypocellularity, sparse vascularity, and abundant myxoid stroma, most commonly arising in large skeletal muscles such as the thigh and shoulder girdle. It typically presents as a slow-growing, painless mass in middle-aged adults and is thought to originate from fibroblastic or mesenchymal cells, with GNAS mutations identified in many cases. Although benign, its clinical and radiological features may mimic low-grade myxoid sarcomas, creating diagnostic challenges. Penile involvement is exceptionally rare, with very few cases reported in the literature, and this unusual location often raises concern for malignant soft tissue tumours. Accurate diagnosis, therefore, relies on histopathological evaluation, supported by imaging, to avoid overtreatment. Complete surgical excision is curative, with recurrence being uncommon, and reporting such cases contributes to improved recognition and management of this rare penile tumour.

This case concerns a 27-year-old man with a slow-growing mass on the ventral aspect of the distal penile shaft, present for over 10 years, which posed a diagnostic dilemma. However, with the aid of imaging and histology, a confirmation of this rare pathology was made.

## Introduction

Intramuscular myxoma (IMM) is a rare benign soft tissue tumour that arises from mesenchymal tissues within skeletal muscle, which lose their capacity to produce collagen but produce excess hyaluronic acid and immature collagen fibres. They occur at various locations, such as the heart, bones, skin, subcutaneous and aponeurotic tissues, the urogenital tract, and skeletal muscles, with the large muscles of the thigh, gluteal region, and upper arm being the most common locations [1]. It is characterised by a hypocellular myxoid matrix containing sparse, spindle-shaped cells and minimal vascularity. While it behaves in a benign manner, its clinical presentation and imaging findings often overlap with those of low-grade myxoid sarcomas, making an accurate diagnosis essential. With advances in molecular pathology, particularly the identification of activating mutations in the GNAS gene, the understanding and diagnostic accuracy of IMM have improved significantly in recent years [2].

## Case presentation

A 27-year-old man presented with a mass on the distal part of his penis. It had been noticed over 10 years earlier and was slow-growing. There was no associated pain or discharge, and it did not cause any lower urinary tract symptoms or erectile dysfunction. There was no swelling in any other part of his body. He had no other comorbidities and no family history of lumps.

Examination findings

**Figure 1:**
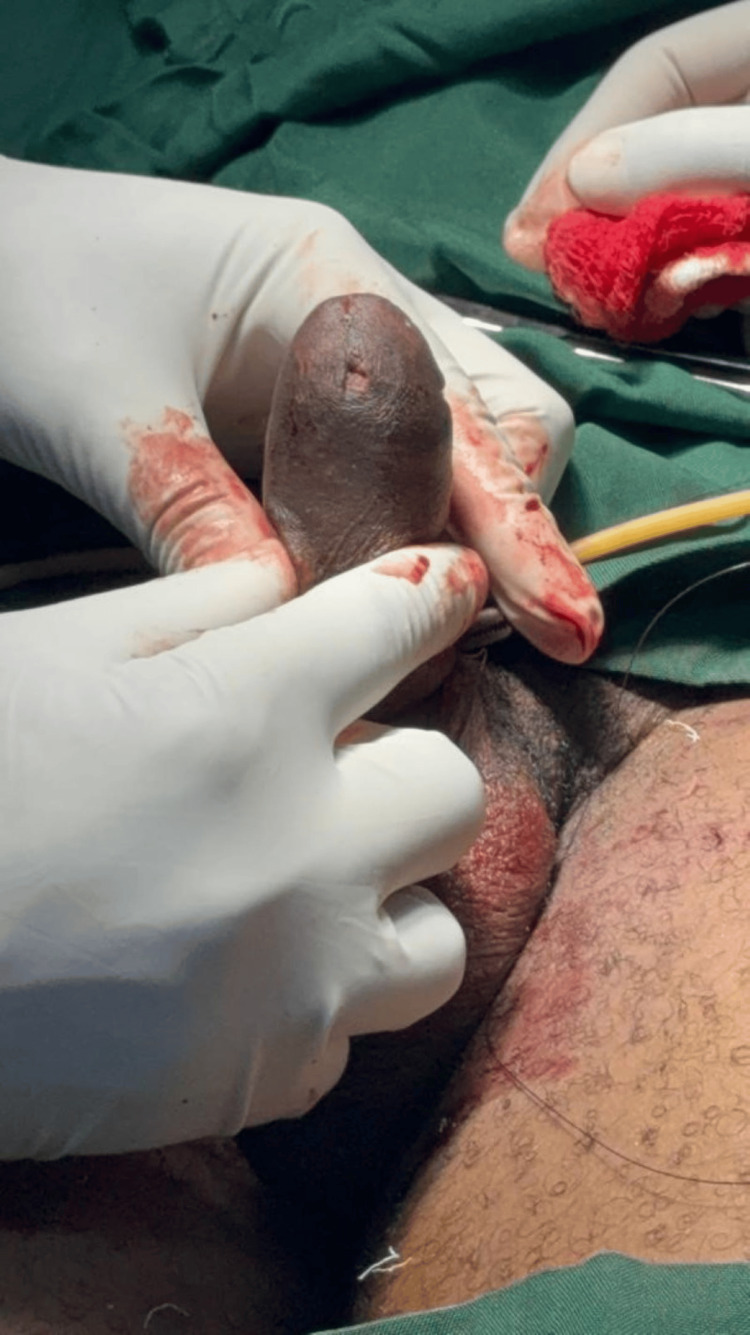
Swelling located on the ventral aspect of the glans penis

Circumcised phallus with multiple skin bridges between the corona of the glans and the distal aspect of the penile shaft. Two mobile masses were palpated at the ventral aspect of the distal penile shaft, just short of the area of attachment of the penile skin bridges. Each of the masses measures about 2 × 1 cm in dimension, and is firm and non-tender (Figure 1).

**Figure 2:**
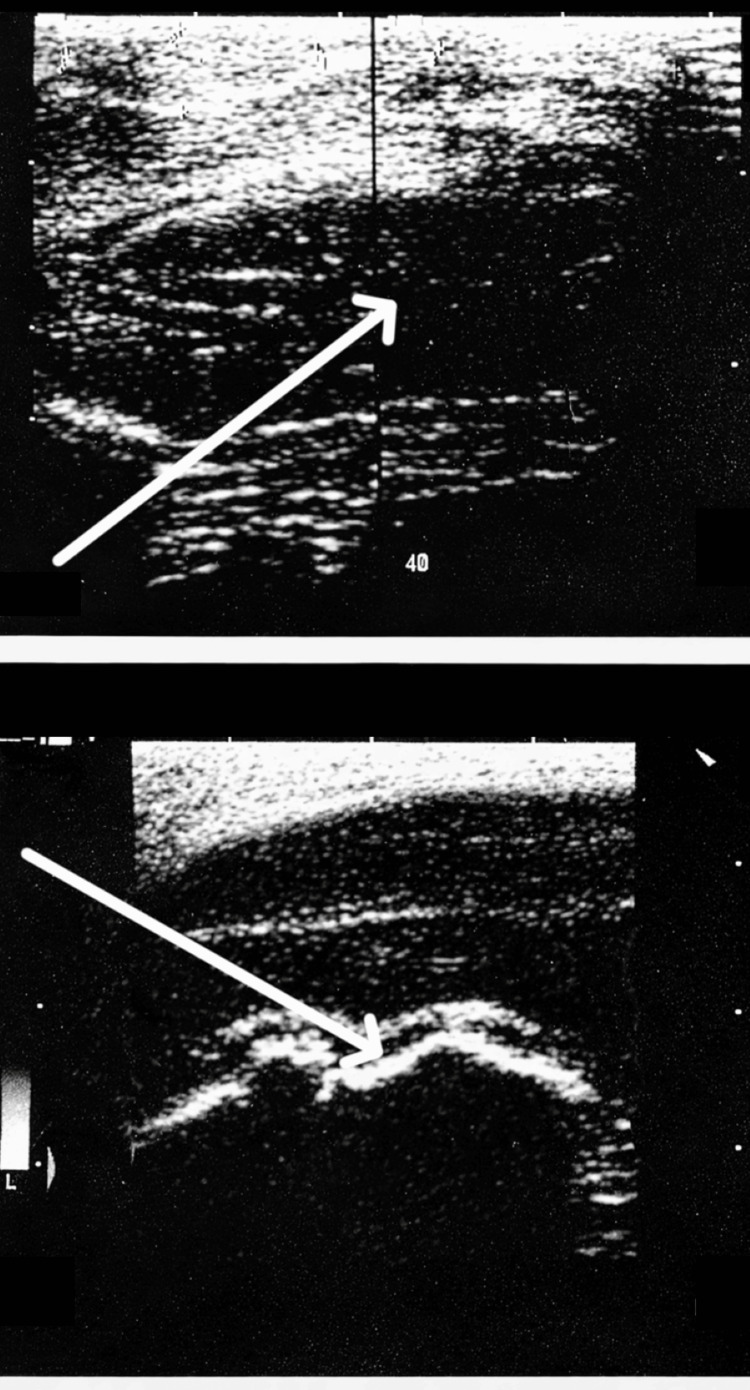
Ultrasound image with arrow showing the lesion

Blood and urine investigations were normal. Ultrasound revealed an oval-shaped, isoechoic mass measuring 5 × 15 × 18 mm in the subcutaneous tissue of the ventral aspect of the glans. Other surrounding tissues appeared normal (Figure 2).

**Figure 3:**
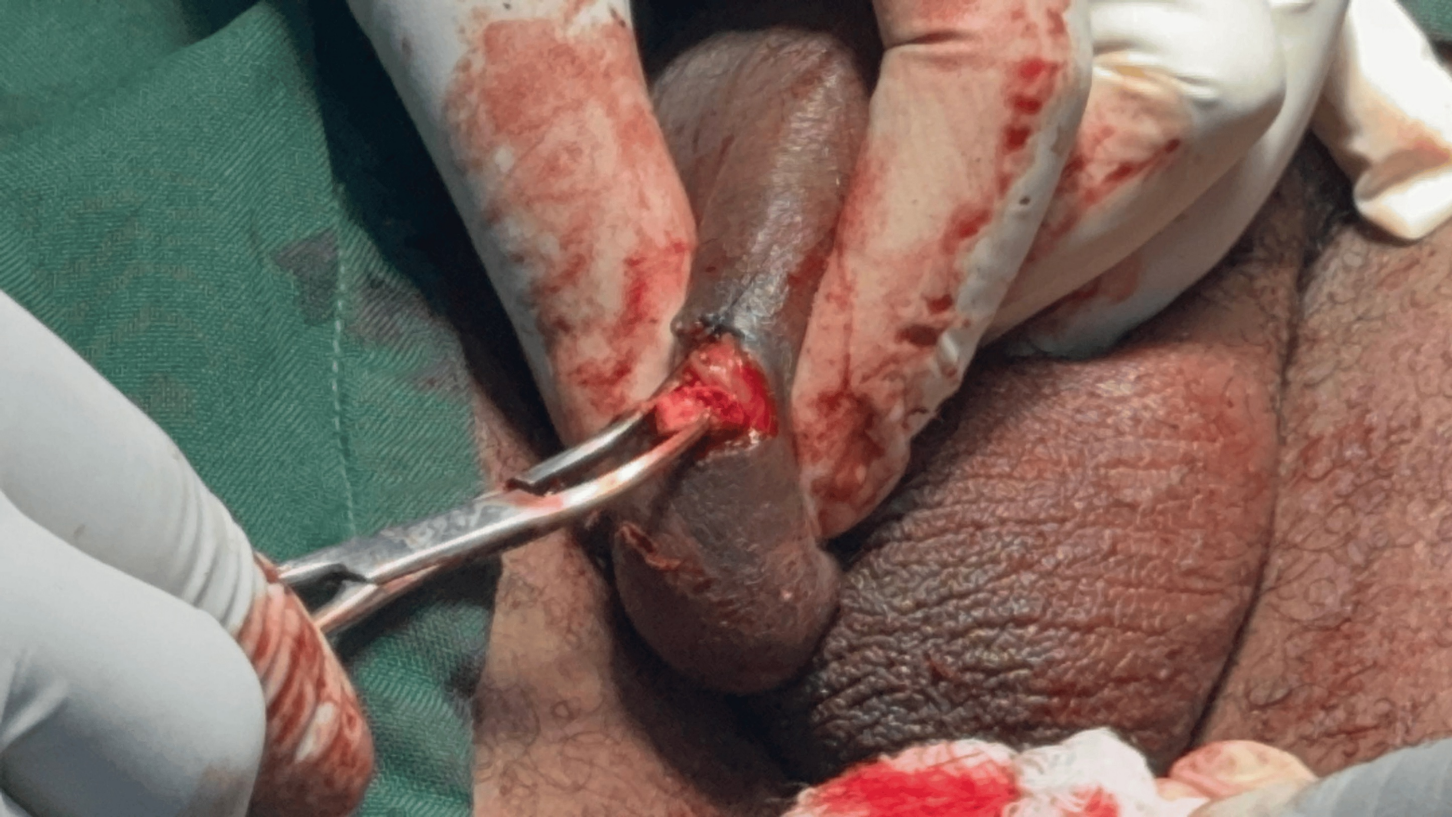
Incision made with excision of myxoma

**Figure 4:**
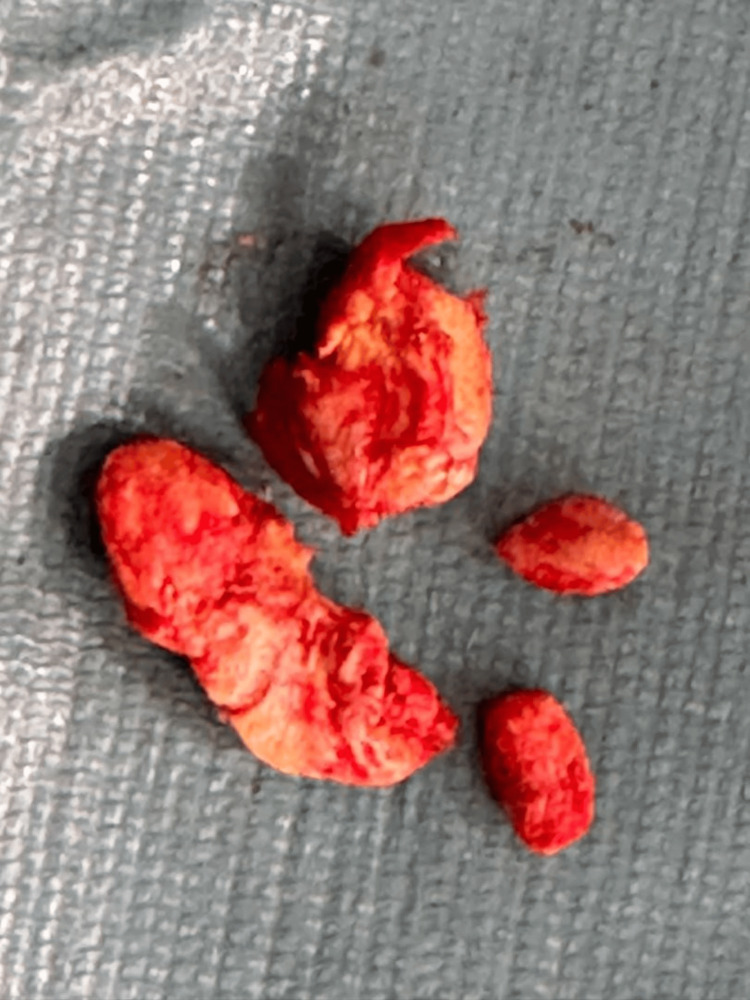
Lumps of tissue following excision

The patient underwent excision of the mass under spinal anaesthesia. Intraoperatively, the swellings were located at the ventral aspect of the distal penile shaft, just short of the glans penis, at the level of the Dartos fascia. Figure 3 shows the excision of the masses, with emphasis on the depth at which the masses were found, while Figure 4 shows the completely excised masses on display.

**Figure 5:**
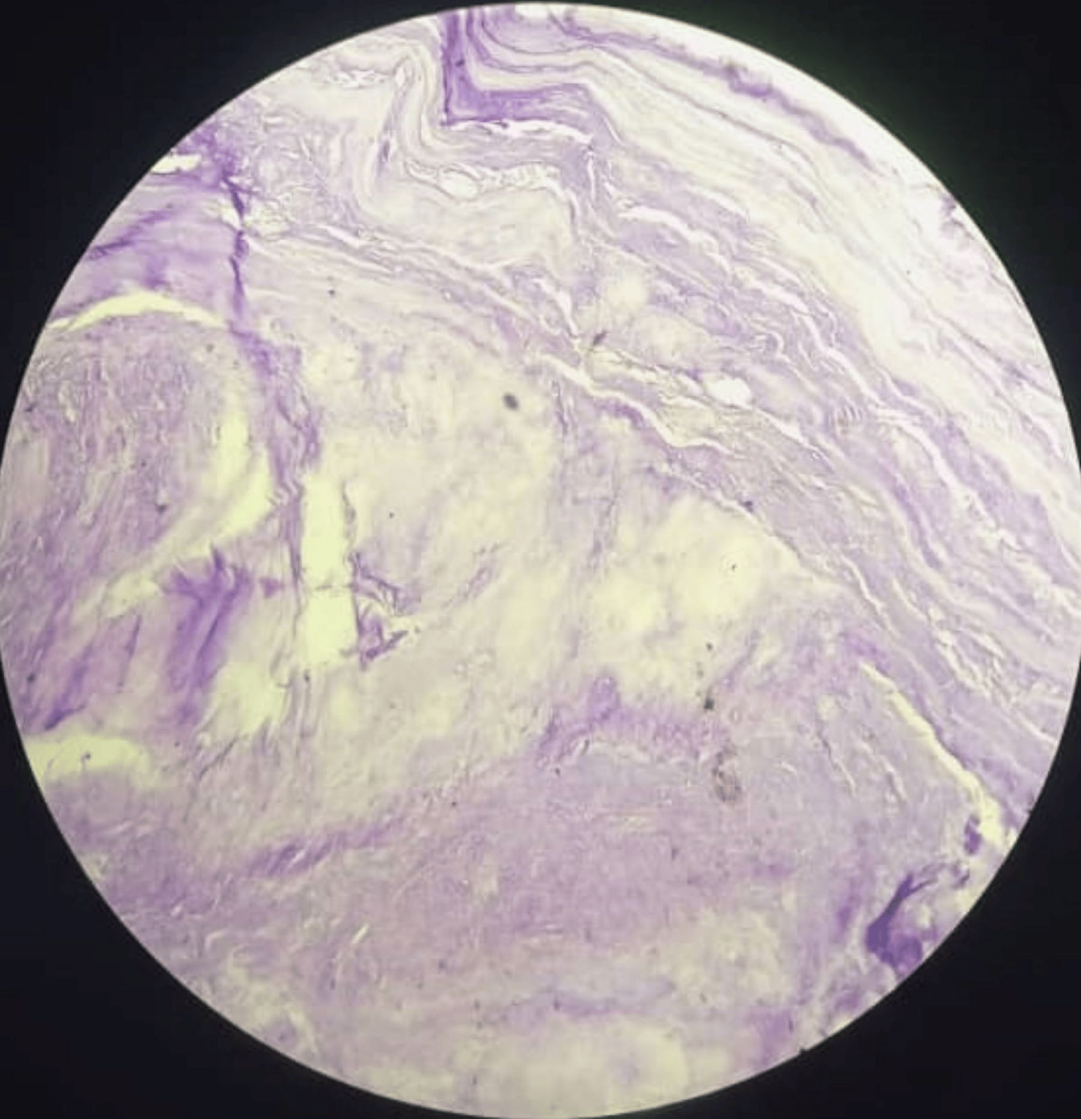
Histology section showing spindle cells in abundant extracellular myxoid stroma

Histology revealed sections of soft tissue with bland, hypocellular lesions containing spindle cells in abundant extracellular myxoid stroma. There were occasional ovoid nuclei without atypia. No inflammation was seen. No atypia was seen. This was in keeping with IMM, as shown in Figure 5.

Postoperatively, the patient was discharged home the next day and was called for follow-up after a period of two weeks.

## Discussion

IMM is a rare benign mesenchymal tumour, with a global incidence estimated at approximately 0.1 to 0.13 per 100,000 persons per year, or roughly one case per million annually [3,4]. This estimate, however, is not based on robust population-level registry data, but rather on retrospective case series and literature reviews. IMM typically presents in adults between the fourth and seventh decades of life and exhibits a female predominance, with women representing up to 60%-70% of reported cases [1,2]. In North America, several case series and reports have documented IMM in typical anatomical sites and demographic groups, consistent with global data. Reports from U.S. institutions reflect the common presentation of IMM in women over 40 years of age, with deep intramuscular masses in the lower extremities [5,6]. In South America, IMM is also sparsely reported. Isolated cases have been documented in Japan, with similar demographic and anatomical characteristics [7]. Similarly, in India, IMM is regarded as rare, with only a few documented cases in the literature [8]. In Europe, the epidemiological profile is similarly based on institutional experiences and case reports, rather than population-level data. Several European studies have examined the imaging features and histological characteristics of IMM, confirming similar age and sex distributions to those in North America [9,10]. Some molecular studies from Austria and the Netherlands have highlighted the prevalence of GNAS mutations in IMM, reinforcing diagnostic criteria, but no continent-wide incidence studies have been conducted [1,2]. In Asia, IMM has been reported in regions such as Europe and Asia, primarily in the form of single case reports or small case series [11-13]. These reports mirror global trends, with IMM presenting in middle-aged women and most commonly in the thigh muscles. Reports from Africa are extremely limited. A small number of case studies from Turkey have described IMM in standard anatomical locations [14].

Clinically, patients present with a slow-growing, painless, deep-seated mass, most frequently involving large skeletal muscles of the thigh, buttock, or shoulder girdle. Smaller muscles, such as those in the hand or paraspinal region, can be affected but are rare [15]. Systemic symptoms are absent, and most patients are otherwise asymptomatic. Due to its indolent nature and benign behaviour, IMM is often underdiagnosed, especially in settings with limited access to advanced imaging and pathology services.

The most critical recent advancement in understanding IMM has been the identification of activating mutations in the GNAS gene, particularly at codon 201 (R201H and R201C), and, less commonly, at codon 227 (Q227E) [1,9]. These mutations result in constitutive activation of the stimulatory G-protein alpha subunit (Gsα), leading to increased intracellular cAMP levels and promoting tumorigenesis. In a 2024 study of 22 IMMs, Hatchett et al. found GNAS mutations in 86% of tumours using Sanger sequencing and AmpliSeq [1]. Additional studies employing more sensitive techniques, such as single-molecule tagged molecular inversion probes (smMIPs), have detected rarer variants, like R201S, R201L, and Q227R [9]. These findings support the utility of GNAS mutation analysis as a diagnostic adjunct. Cytogenetically, some IMMs also exhibit chromosomal gains, especially of chromosomes 7 and 8, but these are non-specific [1].

MRI is the imaging modality of choice in evaluating suspected IMM. Characteristic findings include a well-demarcated intramuscular mass with low T1 and high T2 signal intensity, often surrounded by a rim of fat, and sometimes demonstrating mild to moderate post-contrast enhancement [16]. Histologically, IMM is composed of sparsely cellular, bland spindle or stellate cells within an abundant myxoid matrix, with low mitotic activity and absent nuclear atypia [17]. A cellular variant of IMM exists, with slightly increased cellularity and vascularity, but still benign features [18]. Immunohistochemically, tumour cells typically express vimentin and CD34, but are negative for S-100, desmin, and EMA, which helps differentiate IMM from myxoid neurogenic or sarcomatous tumours [17].

IMM must be distinguished from several histologically similar, but clinically more aggressive, tumours, such as low-grade myxofibrosarcoma, myxoid liposarcoma, and low-grade fibromyxoid sarcoma. These malignant neoplasms tend to show increased cellularity, nuclear atypia, mitoses, infiltrative margins, and specific translocations, such as FUS-CREB3L2 in fibromyxoid sarcoma [17,19]. Importantly, GNAS mutations are not typically found in these malignant counterparts. A recent 2024 study, using DNA methylation profiling, showed that cellular myxomas have distinct methylation signatures from low-grade myxofibrosarcomas, supporting their classification as benign [20].

Surgical excision, with marginal or wide local resection, is the treatment of choice for IMM. Recurrence is rare, particularly when the tumour is completely excised [3,21]. No reports of metastasis have been documented. In challenging anatomical locations, microsurgical tools or endoscopic assistance have been used to achieve complete excision while minimising morbidity [22]. For instance, a 2025 case report described the use of an endoscopic microinspection tool (QEVO®) to facilitate safe tumour removal in the lumbar spine [22]. Prognosis following surgery is excellent, and no adjuvant therapy is required. In a study with over 20 years of follow-up, no recurrences or malignant transformations were observed [3].

## Conclusions

IMM is a rare but well-characterised benign tumour with distinct clinical, histopathological, and molecular features. The identification of GNAS mutations in the majority of cases has significantly improved diagnostic accuracy. Imaging findings, especially MRI, are helpful but not definitive; histological evaluation remains the cornerstone of diagnosis, and immunohistochemistry is an added advantage. Surgical excision offers an excellent prognosis, with minimal risk of recurrence. Continued research into molecular markers, methylation profiles, and tumour behaviour will further enhance our understanding and management of this unique entity.
